# Integrated Approach to Cyclopiazonic Acid Cytotoxicity Using *In Vitro* (2D and 3D Models) and *In Silico* Methods

**DOI:** 10.3390/toxins16110473

**Published:** 2024-11-03

**Authors:** Carmen Martínez-Alonso, Luana Izzo, Yelko Rodríguez-Carrasco, María-José Ruiz

**Affiliations:** 1 Department of Preventive Medicine and Public Health, Food Science, Toxicology and Forensic Medicine, Faculty of Pharmacy and Food Science, University of Valencia, Av. Vicent A Estelles s/n, Burjassot, 46100 Valencia, Spain; carmen.martinez-alonso@uv.es (C.M.-A.); m.jose.ruiz@uv.es (M.-J.R.); 2 Department of Pharmacy, University of Naples “Federico II”, Via Domenico Montesano, 49, 80131 Naples, Italy; luana.izzo@unina.it

**Keywords:** spheroids, cytotoxicity, cyclopiazonic acid, SH-SY5Y cells, *in silico* method

## Abstract

Cyclopiazonic acid (CPA) is an indole-tetramic acid neurotoxin produced by *Aspergillus* and *Penicillium* genera present mainly in fruit, cereals and nuts. This study compares the cytotoxicity produced by CPA after 24, 48 and 72 h of exposure using both monolayers and 3D spheroids in human neuroblastoma SH-SY5Y cells. Furthermore, CPA toxicokinetics was evaluated using *in silico* models. Cytotoxicity increased dose- and time-dependently, as shown by the MTT assay. The lowest CPA IC_50_ values were found in the monolayer study compared to the 3D spheroids at all exposure times (24 h: 864.01 vs. 1132; 48 h: 437 vs. 1069; 72 h: 392 vs. 567 nM). The CPA exposure on SH-SY5Y spheroid organization and morphology was also studied. Morphological changes, including spheroid disaggregation, were observed after mycotoxin exposure. The *in silico* methods, SwissADME and admetSAR, were used for short and full ADMEt profiles of CPA. The ADMEt predictive profile shows high gastrointestinal absorption and ability to penetrate the blood–brain barrier. Including *in silico* studies emphasizes the comprehensive approach to understanding mycotoxin toxicity and risk assessment. By combining *in vitro* 3D spheroid models with computational simulations, this study aims to provide a holistic perspective on the effects of CPA, enhancing the accuracy and relevance of our findings.

## 1. Introduction

Mycotoxins, a diverse and heterogeneous group of toxic secondary metabolites produced by various species of filamentous fungi, represent a growing concern for both human and animal health [1]. The main mycotoxin-producing fungi belong to the genera *Aspergillus*, *Penicillium*, *Fusarium*, *Claviceps* and *Alternaria*. These compounds can contaminate food and feed, leading to health risks and significant economic losses due to the rejection of contaminated commodities. In fact, mycotoxins are consistently among the top three most reported hazard categories in the Rapid Alert System for Feed and Food (RASFF). According to the latest RASFF publications, there were 485 notifications related to mycotoxins, representing a 10.5% increase compared to the previous year [2]. Given the growing concern over mycotoxins and their substantial impact on both human and animal health, this study focuses on a cyclopiazonic acid (CPA), known for its potential toxicological effects.

The ergot-like alkaloid CPA is an indole-hydrindane-tetramic acid neurotoxin. It is produced by certain fungal species, belonging to the *Penicillium* and *Aspergillus* genera. The CPA has attracted interest in recent years because of its potential toxicological effects on humans and animals. Its presence has been detected in a wide range of foods including cheeses, figs, maize, rice, peanuts, millet, chicken meat and feeds, raising a concern for food safety [3]. Poultry are well known to be affected by CPA, and it is believed that it could be involved in the original out-break of Turkey X disease, which led to the discovery of aflatoxins [4]. CPA can cause a variety of symptoms, depending on species. Symptoms may involve weight loss, diarrhea, muscle and internal organ degeneration and necrosis, which can lead to convulsions that result in death [5]. CPA exerts its toxic effects by inhibiting calcium-dependent ATPases and disrupting calcium homoeostasis [6,7]. Prolonged exposure to CPA may produce degenerative diseases like nephrotoxicity, hepatotoxicity and neurotoxicity [8].

Assessing the risk of mycotoxins through in vivo studies is both costly and time-consuming. Hence, there is a need for higher throughput, cost-effective alternative methods to animal experiments to inform about risk-based safety decisions. In this context, the scientific assessment released by the European Food Safety Authority (EFSA) indicates that *in silico* methods have demonstrated an enhanced ability to perform precise risk assessments, especially in the field of predictive toxicology [9]. *In silico* toxicology methods analyze and predict the absorption, distribution, metabolism, excretion and toxicity (ADMEt) of chemical products based on their chemical structure, often in conjunction with other toxicity assays, drawing from experimental data and quantitative structure–activity relationships [10]. Computational approaches in toxicology can significantly increase our comprehension of the molecular and biological basis of the toxicokinetic mode of action, leading to increased confidence in extrapolations. These methods also identify crucial factors for risk assessment, such as notable susceptibility elements, at-risk subpopulations, and impacts on dose–response curves. Variability in ADMEt process can influence the toxicity profile of substances like mycotoxins, with metabolism playing an essential role. *In silico* models enable the assessment of CPA toxicokinetics. The SwissADME and admetSAR (version 3) open-source software models are reliable, quick and user-friendly, providing a credible substitute for experimental methods [11,12].

Traditionally, toxicity studies have been performed in two-dimensional (2D) monolayer cultures. The 3D spheroid cellular models represent an important step forward toxicity testing compared to traditional 2D *in vitro* models. The physiological relevance of spheroids is due to the capacity to simulate the complex architecture of natural tissues and organs, allowing a more accurate representation of in vivo microenvironments compared to 2D models [13]. They enable researchers to study complex cell–cell and cell–matrix interactions, closely resembling the natural tissue structure. These models offer a more realistic representation of how substances interact with target cells and tissues, leading to more reliable predictions of efficacy and toxicity. Moreover, they present capacity to bridge the gap of the traditional *in vitro* models that lack multicellular structure communication associated with the neuronal system in vivo [14,15,16].

In the last decade, different 3D culture methods have been developed, including well-established spheroids, organotypic cultures, organoids, organ-on-a-chip systems and 3D-printed tissue. To date, the spheroid-based model stands out as the most extensively studied and commonly used as a 3D system. This is mainly due to its straightforward standardization, the enhanced interactions between cells and between cells and the matrix, which support greater differentiation, more realistic cell division rates, the recovery of function that was lost in 2D models, increased resistance to xenobiotic treatments and physiological distribution of the medium’s nutrients and growth ingredients [17].

Our investigation focuses on human neuroblastoma (SH-SY5Y) cells, a well-established cell line frequently used in toxicological studies. To the best of our knowledge, this is the first research that investigates the potential toxicological effects of CPA in SH-SY5Y monolayer cells and 3D spheroids. The aims of this work were: (i) to predict the different physicochemical properties and ADMEt parameters of CPA through *in silico* models, (ii) to check for optimal SH-SY5Y spheroid production and (iii) to compare the cytotoxic effects of CPA on SH-SY5Y monolayer cells and 3D spheroids.

## 2. Results

### 2.1. SH-SY5Y Spheroids Cultures

**Figure 1:**
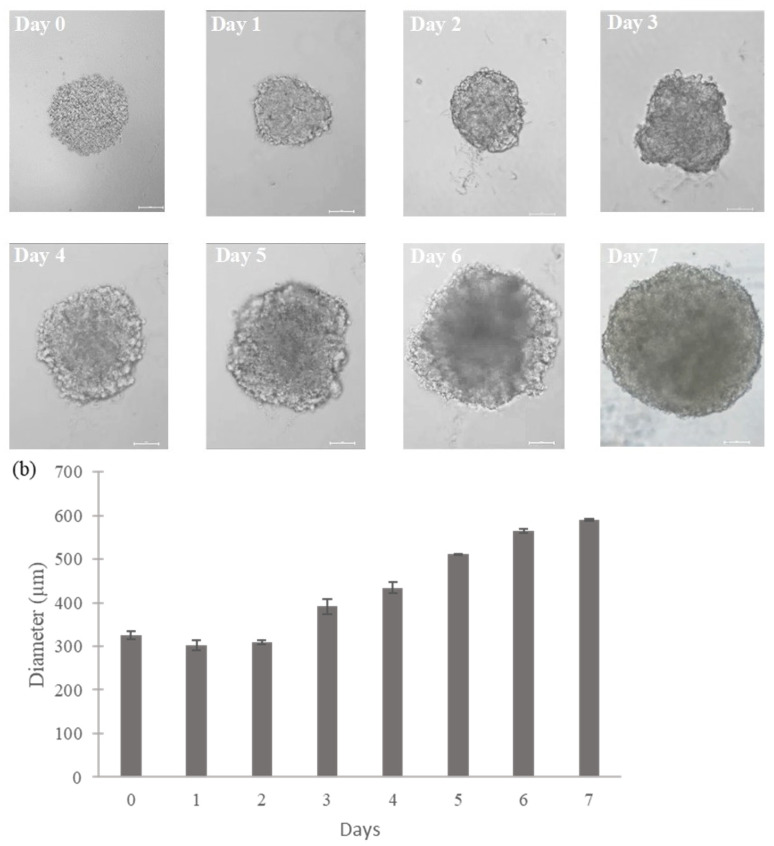
SH-SY5Y spheroids growth in ULA 96-well round-bottom plates. (**a**) Bright-field images of SH-SY5Y spheroids growth from 0 to 7 days. Images were obtained using the Light Microscope Zeiss Axio Observer (Zeiss Microscopy, Oberkochen, Germany) at 10× magnification. Scale bars: 100 μm; (**b**) Diameter of SH-SY5Y spheroids plotted over time (7 days). Values are expressed as means ± SD (*n* = 3 spheroids/timepoint). Diameters were calculated using Zen Lite version 3.8 software.

Spheroids originating from the SH-SY5Y cell line rapidly developed distinct structures that were centrally located in each well of the plates within a single day. The initial seeding density was determined to achieve an average spheroid size of approximately 550 μm in diameter. This dimension is considered suitable for the natural development of oxygen and nutrient gradients, as well as varying proliferation rates, which are crucial for meaningful 3D experimental research. The development of these spheroids over time is shown in Figure 1a. Notably, spheroids exhibited a consistent increase in size while maintaining their spherical morphology, reaching a desired diameter by day 6–7 (Figure 1b). Furthermore, on day 7, the spheroids exhibited a SI of 0.99, as calculated using Equation (1). Therefore, spheroids of this size can be considered mature, demonstrating regular and spheroidal shapes and being appropriate to carry out experimental assays such as cytotoxicity assays [17,18]. Consequently, day 7 was chosen as the reference point for starting the experiments (time 0).

### 2.2. Cytotoxic Effects of CPA Exposure in SH-SY5Y Monolayer Cultures

**Figure 2:**
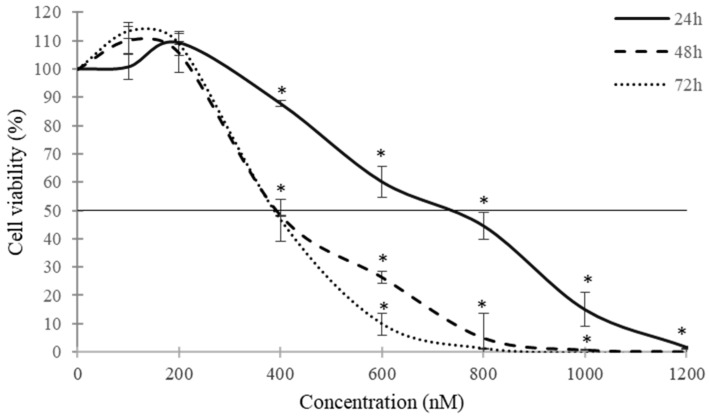
Cytotoxic effects of CPA in SH-SY5Y monolayer cultures obtained by MTT assay after 24, 48 and 72 h of exposure. Data are expressed as mean ± SEM of three independent experiments (*n* = 3). (*) *p* ≤ 0.05 indicates a significant difference compared to the control.

The cytotoxic effects of CPA in SH-SY5Y monolayer cultures were evaluated by the MTT assay after 24, 48 and 72 h. Cells exposed to CPA (from 100 nM to 1200 nM of CPA) decreased cell viability in a time- and concentration-dependent manner (Figure 2). CPA showed significant cytotoxic effects from 400 nM, with a viability reduction of 12%, 52% and 53% after 24, 48 and 72 h of exposure, respectively. The highest cell death was achieved at 800 nM of CPA after 48 and 72 h of exposure and at 1200 nM of CPA after 24 h of exposure. The IC_50_ values obtained by the MTT assay ranged from 864.01 ± 164.09 to 392.33 ± 10.95 nM after the applied exposure times (Table 1).

### 2.3. Cytotoxic Effects of CPA Exposure in 3D SH-SY5Y Spheroids

**Figure 3:**
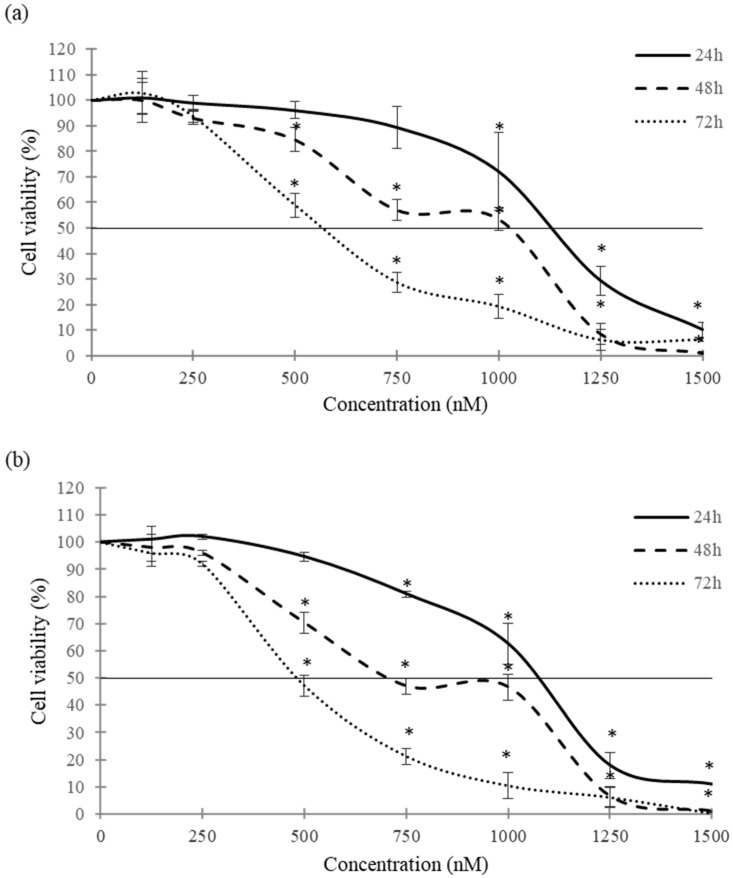
Cytotoxic effects of CPA in 3D SH-SY5Y spheroids by MTT (**a**) and ATP (**b**) assays. Data are expressed as mean ± SEM of three independent experiments (*n* = 3). (*) *p* ≤ 0.05 indicates a significant difference with respect to the control.

The cytotoxic effects of CPA (125–1500 nM) in SH-SY5Y spheroids after 24, 48 and 72 h of exposure were determined by ATP and MTT assays. The CPA concentration range was selected considering the previously IC_50_ values obtained in 2D monolayer cultures, and the lower sensitivity of 3D cultures compared to SH-SY5Y monolayer cultures [17,19]. Spheroids exposed to CPA revealed a decrease in cell viability in a time- and concentration-dependent manner by both methods (Figure 3). Results shown in Figure 3a demonstrated a significant cytotoxic effect at 500 nM CPA, resulting in a 15% and 41% cell viability reduction after 48 and 72 h of exposure, respectively. The IC_50_ values obtained through the MTT assay after 24, 48 and 72 h of exposure to CPA ranged from 1132.37 ± 46.33 to 567.22 ± 34.42 nM (Table 1). Similar results were obtained by an ATP assay, where the IC_50_ values obtained were 1061.87 ± 61.81, 836.69 ± 129.13 and 493.12 ± 48.82 nM after 24, 48 and 72 h of exposure, respectively. The ATP assay also showed a decrease in cell viability at 500 nM CPA, up to 30% and 53% in 3D spheroids after 48 and 72 h of exposure, respectively (Figure 3b). The raw data supporting the analysis are available in the File S1(RawData_ATP_MTT_ACP_3D.xlsx).

Table 1 summarizes the IC_50_ values obtained by the MTT assay in SH-SY5Y cells in 2D and 3D experiments after CPA exposure at the three different times of exposure.

### 2.4. Analysis of 3D SH-SY5Y Spheroids Exposed to CPA

**Figure 4:**
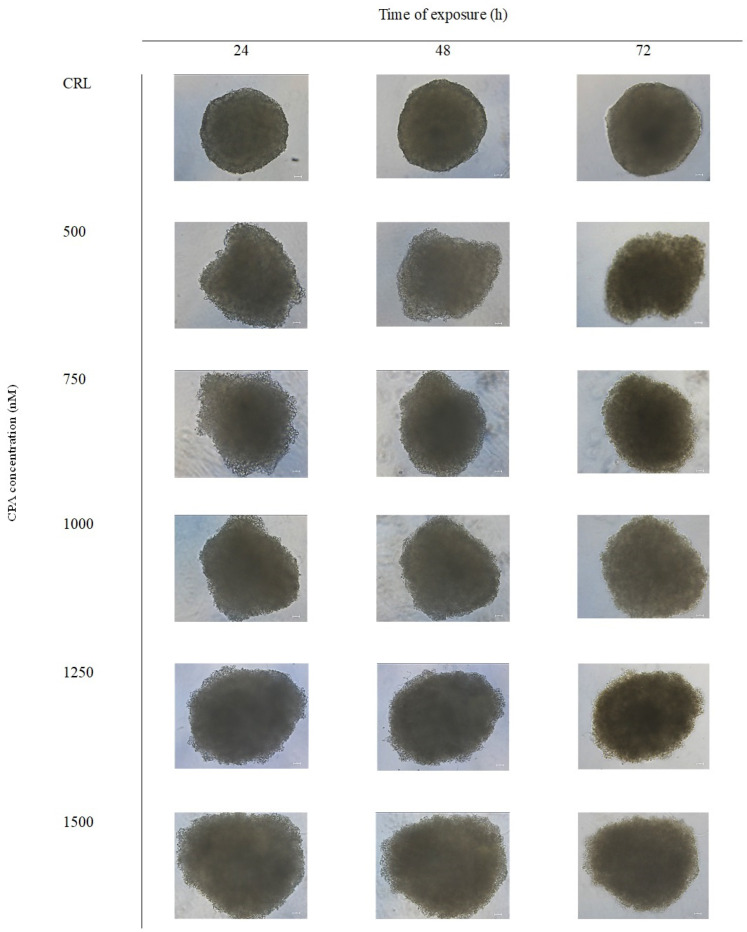
Bright-field images of SH-SY5Y spheroids after 24, 48 and 72 h of exposure to increasing concentrations of CPA (500–1500 nM). Spheroids exposed to DMSO (≤1%) were used as control (CRL). Scale bar: 50 μm. Images were obtained using the Light Microscope Zeiss Axio Observer (Zeiss Microscopy, Germany).

In this work, we determined the effects of CPA on spheroid organization and morphology. To achieve this objective, bright-field images of the spheroids were captured after 24, 48 and 72 h of exposure to CPA, followed by 3D image analysis. Figure 4 shows the morphology of SH-SY5Y spheroids after CPA exposure at all the assayed times. Morphological examination revealed that CPA exposure led to a progressive disaggregation of the spheroids, losing their structure at all tested concentrations and different times of exposure. Following exposure to CPA, the SH-SY5Y spheroids exhibited a loss of compactness, manifesting as an irregular surface appearance. This contrasts with the compact structure observed in the control spheroids (Figure 4).

**Figure 5:**
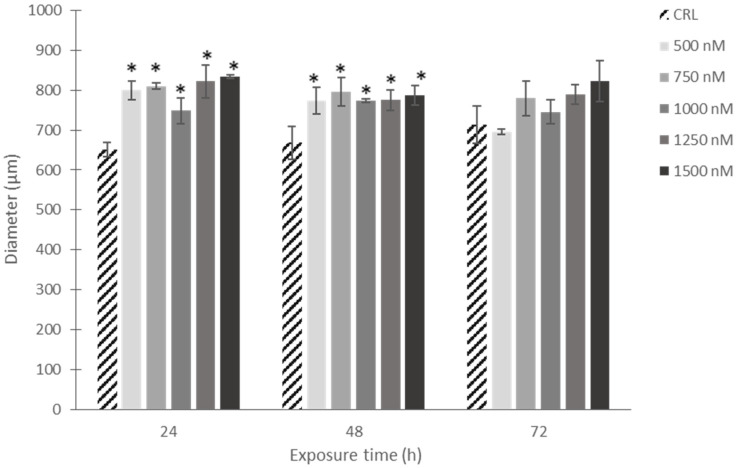
Diameter of SH-SY5Y spheroids after CPA (500–1500 nM) exposure for 24, 48 and 72 h. Quantitative analysis was performed using the software Zen Lite version 3.8 (Zeiss Microscopy, Germany). Results are expressed as the mean ± SEM of four independent spheroids for each concentration and time (*n* = 4). CRL: control. (*) *p* ≤ 0.05 indicates a significant difference compared to the control.

The visual observation under the microscope showed the effect of increasing concentrations of CPA on the reduction in compactness, which resulted in an increase in spheroid size (diameter). This was corroborated by quantitative analysis using the software Zen Lite version 3.8. These results indicated a significant increase in the diameter of the spheroids induced after CPA exposure at all concentrations tested after 24 and 48 h of exposure compared to the control. However, after 72 h of CPA exposure the spheroids’ diameters did not differ from the control spheroids at any concentration tested (Figure 5).

### 2.5. In Silico ADMEt Profile Prediction

The fundamental ADMEt profile prediction of the CPA is shown in Table 2. According to Lipinski’s Rule of Five, CPA typically exhibits favorable absorption or permeation following oral exposure [20]. The predictive tool indicates that CPA is a substrate of the CYP3A4 and CYP2C9 isoenzymes (Table 2), which are crucial in Phase I oxidative metabolism. But it is also an inhibitor of CYP2C9 and CYP1A2. Additionally, CPA is a substrate for metabolic clearance mediated by enzymes other than CYPs, predominantly UDP-glucuronosyltransferases (UGTs).

In accordance with ADMEt profile prediction, CPA toxicity is manifested at the mitochondrial level, which is corroborated by the findings of the MTT and ATP assays, both related to mitochondrial activity.

Regarding mycotoxin transporters, the admetSAR tool suggests that CPA inhibits transporter activity (Table 2). Specially, CPA has been identified as an inhibitor of organic anion-transporting polypeptides OATP1B1 and OATP1B3. These membrane proteins are primarily located in the liver, kidneys and intestine, where they facilitate the uptake and transport of various endogenous and exogenous organic anions, thereby playing a crucial role in drug metabolism and disposition. Furthermore, CPA inhibits the BSEP transporter, which is critical for bile acid secretion from hepatocytes into bile in humans. It may, therefore, be implicated in cholestasis or other hepatobiliary disorders. Additionally, CPA induced kidney injury and exhibited respiratory and reproductive toxicity. It also has the capacity to bind to estrogen, androgen and glucocorticoid receptors.

CPA, with an *in silico* predicted LD_50_ of 3.39 mol/kg, is expected to be classified in Category 1 under the Globally Harmonized System (GHS) for acute oral toxicity, indicating a high level of toxicity (Table 2). Genotoxicity assays, including the Ames test and micronucleus assay, returned positive results for CPA.

**Figure 6:**
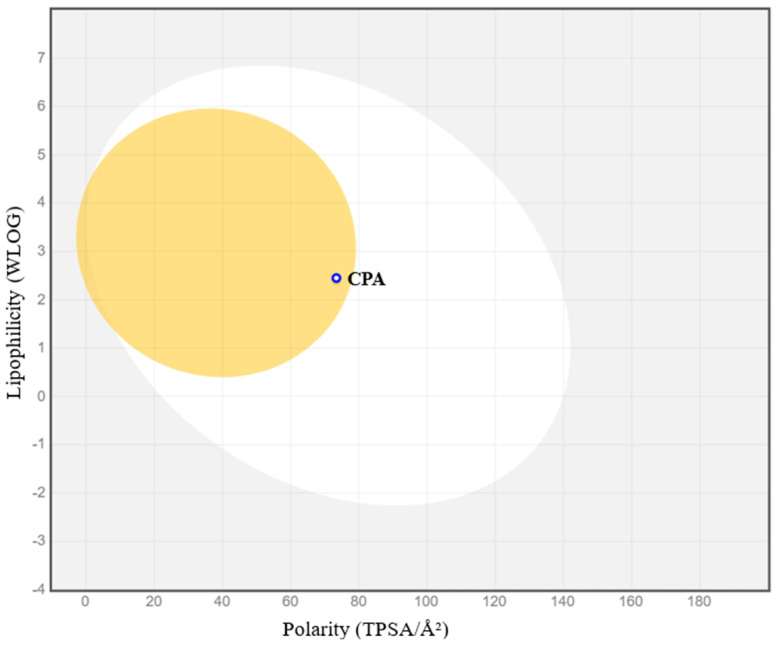
BOILED-Egg predictive model constructed using the CPA SMILES. The ‘yolk’ area represents the physicochemical space where molecules are likely to penetrate the blood–brain barrier (BBB), while the white region denotes the physicochemical space where molecules are likely to be absorbed by the gastrointestinal tract (HIA). The grey area indicates low permeability and absorption. CPA is represented as a single point based on its specific lipophilicity and polarity values. The blue circle indicates that CPA is probably a substrate of P-glycoprotein.

CPA demonstrated high gastrointestinal absorption based on Caco-2 cell permeability and HIA probability. Moreover, CPA is well absorbed according to its Log P_o/w_ (2.45) and TPSA/Å2 (73.4) parameters. These results indicate that the analysis of the BOILED-Egg model, derived from the SwissADME tool, highlights the high probability of gastrointestinal and brain absorption of CPA (Figure 6).

## 3. Discussion

In order to enhance the risk assessment of less studied mycotoxins, more precise safety thresholds need to be established to cover a large number of them [21]. For this reason, understanding their mechanisms of action (MoA) is crucial, given their potential toxicity and adverse effects on living organisms [22]. In this study, the cytotoxicity of CPA was evaluated due to the increasing awareness of its effects and because it is a non-regulated mycotoxin, stemming from the limited data regarding its occurrence in foodstuffs, and its toxicity represents a significant limitation in the available evidence base.

Several authors have reported the toxicity of CPA in various species, including rats, mice, guinea pigs, pigs, monkeys and chickens, with key target organs being the gastrointestinal tract, liver, kidneys, skeletal muscles and the nervous system [23]. In rodents, CPA primarily affects muscle, hepatic tissue and the spleen, with more pronounced toxic changes observed in the liver and spleen [24]. The oral LD_50_ is reported to be in the range of 30 to 70 mg/kg, indicating a moderate level of toxicity. Pigs, which appear to be the most sensitive species, have a no-observable-effect level (NOEL) estimated at approximately 1.0 mg/kg/day. These findings highlight the potential risks associated with CPA exposure across different animal species, indicating a significant toxic response as observed in our study. In this work, the cytotoxic effects of CPA were investigated using two different culture models: 2D monolayers and 3D spheroids. The analysis of spheroid morphology is emerging as a highly dependable and cost-effective approach to assess the cytotoxic effects of treatments with minimal experimental manipulation [25]. Since changes in spheroid volume and area are often related to toxic responses after exposure to cytotoxic compounds [26,27], our findings revealed that CPA induced cytotoxic effects, which were associated with an increase in spheroid size (diameter). Similarly, Zingales et al. (2024) [28] observed significant morphological changes in BM-MSC spheroids exposed to the highest concentrations of patulin (PAT), exhibiting a larger and more relaxed appearance compared to control spheroids. This alteration could be associated with a reduction in cell–cell interactions and the loss of adherents junctions, aligning with previous findings reported by Celli et al. (2014) [29]. As far as the authors are aware, there is no evidence from SH-SY5Y cell-based studies that can be used to determine the cytotoxic effects of CPA. Our results demonstrate that CPA decreases cell viability in both 2D and 3D models. CPA showed significant cytotoxicity effects from 400 nM after 24 and 48 h of exposure in SH-SY5Y monolayer cultures with IC_50_ values of 864.01 ± 164.09 and 436.73 ± 22.12. Hymery et al. (2014) [5] indicated that in human monocytic leukemia (THP1) cells, cytotoxic effects of CPA were evident at concentrations from 44.5 nM and 0.00445 nM after 24 and 48 h of exposure, respectively. Following exposure of 125 nM CPA, a reduction in cell viability was observed, with a decrease of 69% and 59% after 24 and 48 h of exposure, respectively. No IC_50_ value was observed after 24 h of exposure to CPA, whereas an IC_50_ value of 175 nM was observed after 48 h. Moreover, CPA also showed cytotoxic effects in Caco-2 cells at 125 nM and 4.45 nM after 24 and 48 h of exposure, respectively. While our findings confirm the cytotoxicity of CPA, they also indicate that SH-SY5Y cells exhibit reduced sensitivity compared to other cell lines. Furthermore, given the paucity of data on the cytotoxic effects of CPA in SH-SY5Y cell cultures, it is essential to gather information on other mycotoxins produced by the same fungal genera, specifically *Aspergillus* and *Penicillium*. In this context, Mitchell et al. (2024) [30] reported IC_50_ values of 2.01 μM and 1.5 μM after 24 and 48 h of exposure of SH-SY5Y cells to PAT. Similarly, Abudayyak et al. (2023) [31] reported an IC_50_ value of 250.90 μM after 24 h of exposure of the same cell line to citrinin (CIT).

In addition to traditional monolayer cultures, we employed spheroids as an advanced alternative *in vitro* model to assess the cytotoxic effects produced by CPA. We optimized the seeding density and the protocol for spheroid generation to obtain reliable results, which represent the main goal of this study. Our results demonstrated that CPA exhibits significantly lower IC_50_ values in SH-SY5Y spheroids compared to the hepatic spheroids previously studied by Ma et al. (2022) [32]. Specifically, we found that the IC_50_ values in our experimental conditions were 1132.37 ± 46.33 nM, 1069.02 ± 278.76 nM and 567.22 ± 34.42 nM after 24, 48, and 72 h of CPA exposure, respectively. In contrast, Ma et al. (2022) [32] reported IC_50_ values of 231.97 µM, 145.99 µM and 62.36 µM in hepatic spheroids after 24, 48 and 72 h of CPA exposure, respectively. These findings suggest that SH-SY5Y spheroids are more sensitive to CPA than hepatic spheroids, indicating potential differences in cellular response and underscoring the importance of considering cell type in evaluating the toxicity of this compound. Additionally, our results demonstrated IC_50_ values for CPA in both monocultures and spheroids. The results obtained in 2D models suggest that CPA is more cytotoxic than other mycotoxins. Regarding spheroids, SH-SY5Y spheroids exhibited a higher cytotoxic effect compared to that observed in hepatic spheroids [30,31,32]. The hepatic spheroids used in the study by Ma et al. (2022) [32] represent a distinct cellular model compared to SH-SY5Y spheroids. Hepatic spheroids are derived from primary hepatocytes, which possess unique physiological and metabolic traits, while SH-SY5Y cells are neuroblastoma-derived. Although primary cells more accurately reflect in vivo conditions, they are costly, highly variable and labor-intensive. This has led to growing interest in developing cost-effective, scalable, and reproducible cell models that enhance experimental throughput. Such innovations in *in vitro* alternatives support the 3Rs strategy (reduction, refinement, and replacement), promoting reproducibility while reducing reliance on animal testing [33].

As expected, significant differences in IC_50_ values can be observed between the two culture models for all exposure times tested. The SH-SY5Y spheroids showed significantly increased resistance to the cytotoxic effects of CPA exposure compared to monolayer cell cultures (Table 1). These findings align with the results in the literature, indicating that the sensitivity observed in 2D systems does not directly translate to 3D systems, resulting in higher IC_50_ values in 3D cellular models [19,34,35,36,37]. This difference can be attributed to the presence of pronounced intracellular junctions within 3D spheroids, which simulate physiological barriers, and a dense extracellular matrix (ECM) with small pores that affect xenobiotic transport by reducing their penetration. In contrast, the absence of organization in 2D systems versus 3D models may lead to an overestimation of CPA cellular toxicity [38,39]. Similarly to our results, Zingales et al. (2021) [17] reported variations of up to a magnitude difference in IC_50_ values between 2D monolayers and 3D spheroids. Zingales et al. (2021) [17] demonstrated that SH-SY5Y spheroids exposed to sterigmatocystin (STE) for 24 and 48 h did not show IC_50_ values but exhibited an IC_50_ value of 5.18 ± 3.14 μM at 72 h of exposure. In the same line, Csenki et al. (2021) [40] observed that MDCK spheroids were less vulnerable than MDCK monolayer cultures to ochratoxin B (OTB), which is produced by the fungi *Aspergillus* and *Penicillium*. The IC_50_ of OTB in MDCK monolayer cultures was 32.06 µM, while it was greater than 50 µM in spheroids.

In order to determine the toxicokinetic profile of CPA, we used the SwissADME and admetSAR web tools for an *in silico* investigation to determine chemical specific parameters of CPA. We selected these tools due to their accessibility (free access) and the reliability of their computational techniques in estimating the parameters for determination of the *in vitro* toxicity of small molecules. Moreover, these methodologies have been extensively validated with experimental data [13,41,42,43]. Our *in silico* analysis suggests that CPA behaves similarly to bioavailable mycotoxins when orally consumed, indicating its ready absorption within the human intestinal tract and its ability to penetrate the BBB. While there are no *in vitro* or in vivo bioavailability data related to CPA, the predictions obtained in this work are similar to those obtained with other mycotoxins produced by *Penicillium* and *Aspergillus* [44]. Predictive data indicate that CPA serves as a substrate for CYP3A4, which is responsible for metabolizing over half of all therapeutic medications. This enzyme is predominantly found in the liver and intestines, representing around 30% of the total hepatic P450 protein and 80% of the total P450 content in the small intestine [45]. Additionally, CPA also acts a substrate for the CYP2C9, which ranks among the most abundant CYP enzymes and is responsible for metabolizing over 15% of clinical drugs [46]. While both AdmetSAR and SwissADME agree on the intestinal absorption of CPA, there are notable discrepancies regarding its permeability across the blood–brain barrier (BBB) and its status as a substrate for P-glycoprotein. AdmetSAR predicts that CPA is not permeable to the BBB and is not a substrate for P-glycoprotein, whereas SwissADME predicts that it is permeable to the BBB and interacts with P-glycoprotein. This contradiction highlights the complexity of predicting the pharmacokinetics of CPA and interpreting data from different *in silico* models. Future studies should aim to validate these findings *in vitro* to better understand the mechanisms influencing the bioavailability and neurotoxicity of CPA. In addition, *in silico* models offer potential benefits because they are faster, cheaper, high-throughput, and do not require animal testing. Moreover, they are very useful for providing mechanistic data for mycotoxins with a potential hazard risk. However, there is still a gap between in vivo and alternative models. Therefore, it is necessary to understand when and how computational models can be used.

## 4. Conclusions

*In vitro* cytotoxicity studies show that CPA is highly cytotoxic to SH-SY5Y cells in both 2D and 3D culture models, with a time- and concentration-dependent decrease in cell viability. However, a lower response to the cytotoxic effects of the CPA is evident in the 3D models. These observations may be attributed to the use of a more sophisticated model in toxicity studies, where cells are distributed in three dimensions, mimicking an organ rather than being arranged in a monolayer. This highlights the importance of the microenvironment and 3D structure in toxicity assessment. This study represents a significant step in understanding the cytotoxic effects of CPA in 3D models, bringing *in vitro* 2D studies and in vivo studies closer together. On the other hand, an integrated approach using *in vitro* and *in silico* would be beneficial for CPA toxicological risk assessment. In addition, next-generation physiologically-based kinetic (PBK) modelling, based on *in vitro* and *in silico* methods, has the potential to play a significant role in reducing animal testing and supporting regulatory decisions.

## 5. Materials and Methods

### 5.1. Reagents

The reagent-grade chemicals and cell culture components used, namely Dulbecco’s Modified Eagle’s Medium-F12 (DMEM/F-12), penicillin, streptomycin, trypsin/EDTA solutions, fungizone, phosphate buffered saline (PBS), Fetal Bovine Serum (FBS), methylthiazoltetrazolium salt (MTT) dye and dimethyl sulfoxide (DMSO) were acquired from Sigma-Aldrich (Barcelona, Spain). Methanol (MeOH) and ethyl acetate were purchased from Merck Life Science S.L. (Madrid, Spain). Deionized water (resistivity < 18 MΩ cm) was obtained using a Milli-Q water purification system (Millipore, Bedford, MA, USA).

A standard solution of CPA (≥98% purity) was purchased from Sigma-Aldrich (Barcelona, Spain). An individual stock solution of CPA was prepared in DMSO at appropriate working concentrations and maintained at −20 °C. Final concentrations of CPA in the assay were achieved by adding the culture medium. The final DMSO concentration in the medium was ≤1% (*v/v*).

### 5.2. Cell Culture

SH-SY5Y (ATCC CRL-2266) cells were cultured in monolayer in DMEM/F-12 medium, supplemented with 10% FBS, 0.2% fungizone, 100 U/mL penicillin and 100 mg/mL streptomycin. Incubation conditions were pH 7.4, 5% CO_2_ at 37 °C and 95% air atmosphere at constant humidity. The cells were subcultured commonly twice a week, with a small number of sub-passages (<20) to maintain genetic stability without phenotypic changes that could affect response to treatments or experimental conditions because this could impact the accuracy and reproducibility of the results. SH-SY5Y cells were subcultured following trypsinization, using a 1:2 split ratio. The medium was refreshed every 2–3 days. Mycoplasma absence was regularly verified through the MycoAlert™ PLUS Mycoplasma Kit (Lonza, Rockland, ME, USA).

### 5.3. Spheroid Formation

Spheroid generation from SH-SY5Y cells was performed in accordance with Zingales et al. (2021) [17]. Three-dimensional spheroid cultures were established using single-cell suspensions derived from trypsinized SH-SY5Y cells to ensure the generation of a singular, well-centered, and consistently reproducible spheroid per well; ultra-low attachment (ULA) 96-well round bottom plates (Corning^®^, Corning, NY, USA) were employed. Briefly, 200 μL of cell suspension (2 × 10^3^ cells/spheroids) were dispensed into each well. Then, plates were centrifuged at 1200 rpm for 5 min to induce cell aggregation at the bottom of the well. SH-SY5Y spheroids were cultured for 7 days. On day 4, 50% of the medium was removed and fresh medium was added. The growth conditions and culture media utilized for spheroids were identical to those for monolayer cell culture.

An adequate morphology and size of 3D spheroid ensure higher reproducibility of results. For this reason, assessing morphological parameters such as solidity and the sphericity index is the initial step to minimize bias and select the appropriate spheroid for a specific biological application. According to Santo et al. (2016) [47], spheroids are considered to have a regular shape if their solidity values exceed 0.90. Zanoni et al. (2016) [48] classify spheroids as spherical when their sphericity index (SI) is ≥0.90. The SI was determined using the following equation:

(1) $$SI=\frac{\pi \sqrt{4A/\pi }}{\pi d}$$

where A and d are area and diameter of the spheroid, respectively.

The growth and structure of the 3D spheroid cultures were observed over several days to track alterations in size and form. Bright-field microscope images and morphological analyses of spheroids were conducted using the inverted light microscope Zeiss Primo Vert equipped with a Zeiss camera (Axiocam 208 color, Zeiss Microscopy, Oberkochen, Germany) at 10× magnification. The software Zen Lite version 3.8 (Zeiss Microscopy, Oberkochen, Germany) was used to obtain morphological parameters (area and diameter).

### 5.4. Treatment of Monolayer Cell Culture and Spheroids with CPA

#### 5.4.1. SH-SY5Y Monolayer Cultures

SH-SY5Y cells were cultured in 96-well tissue-culture plates by adding 200 μL/well at a density of 2 × 10^4^ cells/well. After the cells reached 80% confluence, the culture medium was replaced by fresh medium containing different concentrations of CPA (100–1200 nM). Then, plates were incubated in the dark at 37 °C, 5% CO_2_ and 95% air atmosphere at constant humidity for 24, 48 and 72 h.

Cell viability was determined by the MTT assay. The MTT assay is based on the capacity of viable cells to metabolize, via a mitochondrial-dependent reaction, the yellow tetrazolium salt to an insoluble purple formazan crystal. The MTT assay was carried out according to the procedure reported by Ruiz et al. (2006) [49]. In summary, after the mycotoxin exposure, the medium containing CPA was removed and each well received 200 μL of fresh medium containing 50 μL of MTT. The plates were returned to the incubator and kept in darkness during 3 h. Then, the MTT solution was removed and 200 μL of DMSO was added followed by 25 μL of Sorensen’s glycine buffer. The absorbance was measured at 595 nm using an automatic ELISA plate reader (MultiSkanEX, Thermo Scientific, Walthman, MA, USA). Cell viability was represented as a percentage compared to the solvent control (≤1% DMSO). Three independent biological experiments were conducted with eight technical replicates each, and the results were expressed as the mean ± standard error of the mean (SEM) of different independent experiments. The mean inhibitory concentration (IC_50_) values were calculated employing SigmaPlot version 11 (Systat Software Inc., GmbH, Erkrath, Germany).

#### 5.4.2. 3D SH-SY5Y Spheroids

The SH-SY5Y spheroids were exposed to CPA from 125 to 1500 nM. Then, plates were incubated in the dark at 37 °C and 5% CO_2_ for 24, 48 and 72 h.

Cell viability was determined by the MTT and the adenosine triphosphate (ATP) assays. The MTT assay was performed as described by Salehi et al. (2017) [37], with some modifications. Briefly, at the end of each exposure time (24, 48 and 72 h), spheroids were individually transferred to a flat bottom 96-well plate with 100 μL of supernatant and 50 μL/well of MTT solution (5 mg/mL PBS). After 4 h of incubation at 37 °C protected from light, the resulting formazan crystals were solubilized in DMSO (50 μL/well). The absorbance was measured at 595 nm using an automatic ELISA plate reader (MultiSkanEX, Thermo Scientific, Walthman, MA, USA).

The ATP assay is a well-established and widely used method for assessing cellular viability of spheroids. The ATP assay was performed using the luciferin–luciferase bioluminescence reaction. Luciferase enzymes catalyze the conversion of ATP and luciferin into oxyluciferin. The luminescent signal is directly proportional to the amount of ATP present in the sample, a marker for metabolically active cells [50]. In summary, 100 μL/well of culture medium were replaced with medium containing CPA to obtain a final concentration ranging from 500 to 1500 nM. After the mycotoxin exposure, spheroids were transferred to an opaque flat-bottom 96-well plate with 50 μL/well of supernatant. Subsequently, an equal volume of CellTiter-Glo reagent was added to each well and the plate was incubated at room temperature, in darkness, during 30 min. Then, the plate was shaken for 5 min at 250 rpm in darkness. Luminescence was measured at 570 nm using a multimode microplate reader (Biotek Synergy H1; Agilent, GA, USA). Intracellular ATP levels were quantified using the CellTiter-Glo^®^ Luminescent Cell Viability Assay (Promega, Madison, WI, USA), according to the manufacturer’s guidelines.

As for monolayer and spheroids, cell viability was expressed in percentage relative to solvent control (≤1% DMSO). Three independent biological experiments were carried out with four technical replicates each. The IC_50_ values were calculated applying SigmaPlot version 11 (Systat Software Inc., GmbH, Erkrath, Germany).

Bright-field images of spheroids after 24, 48 and 72 h of CPA exposure were taken on an inverted light microscope Zeiss Primo Vert equipped with a Zeiss camera (Axiocam 208 color, Zeiss Microscopy, Jena, Germany) at 10× magnification to observe alterations in their diameter and shape with and without CPA exposure. All images were analyzed using the Zen Lite version 3.8 software to obtain the diameter of SH-SY5Y spheroids.

### 5.5. Toxicokinetics of CPA Using a ADMEToolbox

The ADMET profile of CPA was predicted using admetSAR (version 3), a comprehensive and free tool [13,42,43]. These models describe the partitioning interactions of the CPA in the compartments using mathematical equations. An *in silico* evaluation of the potential absorption of CPA in the intestine was conducted using Lipinski’s rule of five. According to Lipinski et al. (2001), a substance is likely to be well absorbed in the intestine if it has a molecular weight (MW) below 500, fewer than five hydrogen bond donors, fewer than ten hydrogen bond acceptors, and a calculated Log*P* value (the logarithm of the 1-octanol/water partition coefficient; CLog*P*) of less than five. If a substance fails to meet two or more of these criteria, its intestinal absorption is predicted to be low.

Regarding the distribution of CPA throughout the body, the plasma stability, blood plasma ratio, ability to bind to blood plasma proteins and ability to cross the blood–brain barrier were taken into account [51,52,53,54]. Liver, gut metabolism and transporters are considered chemical-specific parameters in the ADME process.

A similar tool, SwissADME, that employs the BOILED-Egg (Brain Or IntestinaL EstimateD permeation predictive model) method to predict human gastrointestinal absorption (HIA) and blood–brain barrier (BBB) penetration was also used. The BOILED-Egg method uses a 2D graphical representation to illustrate how a molecular structure compares to the ideal physicochemical parameters for optimal absorption [55]. It distinguishes between well-absorbed and poorly absorbed molecules based on their lipophilicity and polarity, as described by the Log*P* and the polar surface area (PSA), respectively [56].

### 5.6. Statistical Analysis

Statistical analysis of data was carried out using the SPSS version 2.14 (IBM Corp., Armonk, NY, USA). Data were expressed as mean ± SEM of different independent experiments. A paired sample Student’s *t*-test was conducted, and the differences among groups were evaluated using one-way analysis of variance (ANOVA), which was followed by the Tukey HSD post-hoc test for multiple comparisons. It was set a significance level of 0.05.

## Figures and Tables

**Table 1:** The medium inhibitory concentration (IC_50_) values of CPA in SH-SY5Y cultured as monolayer (2D) and spheroids (3D) after 24, 48, 72 h of exposure by the MTT assay. Data are expressed as mean ± SEM of three independent experiments (*n* = 3).

Time of Exposure (h)	IC_50_ Values (mean (nM) ± SEM)	
	2D	3D
24	864.01 ± 164.09	1132.37 ± 46.33
48	436.73 ± 22.12	1069.02 ± 278.76
72	392.33 ± 10.95	567.22 ± 34.42

**Table 2:** ADMET and toxicity profile prediction of CPA.

	CPA	
	Result	Probability
Lipinski molecular descriptors		
HBA (≤10)	3	
HBD (≤5)	2	
clogP (≤5)	2.14	
MW (≤500 g/mol)	336.38	
n-ROTB (≤10)	1	
Absorption		
HIA	+	0.9855
HOB	−	0.5714
Caco-2 permeability	+	0.5549
Distribution		
BPB		
P-gp substrate	−	0.5342
BBB	−	0.5250
Subcellular localization	Mitochondria	0.6932
Metabolism		
CYP3A4 substrate	+	0.6859
CYP2C9 substrate	+	0.7908
CYP2D6 substrate	−	0.8733
CYP3A4 inhibitor	−	0.7365
CYP2C9 inhibitor	+	0.8252
CYP2C19 inhibitor	−	0.5769
CYP2D6 inhibitor	−	0.8341
CYP1A2 inhibitor	+	0.8318
CYP2C8 inhibitor	−	0.6773
UGT-catalyzed	+	0.6000
OATP2B1 inhibitor	−	0.7142
OATP1B1 inhibitor	+	0.8913
OATP1B3 inhibitor	+	0.9373
MATE1 inhibitor	−	0.7800
OCT2 inhibitor	−	0.9066
BSEP inhibitor	+	0.7380
P-gp inhibitor	−	0.7906
Toxicity		
Rat acute toxicity (LD_50_, mol/Kg)	3.39	
Acute oral toxicity (Category)	1	0.7983
Respiratory toxicity	+	0.7778
Reproductive toxicity	+	0.7333
Mitochondrial toxicity	+	0.7875
Nephrotoxicity	+	0.5974
Estrogen receptor binding	+	0.5289
Androgen receptor binding	+	0.6231
Thyroid receptor binding	−	0.5000
Glucocorticoid receptor binding	+	0.5591
Micronucleus assay	+	0.7700
AMES mutagenesis	+	0.8700
Carcinogenesis	−	0.9000

The “+” sign indicates a favorable prediction for the respective factor, while the “−” sign indicates an unfavorable prediction.BBB: blood–brain barrier; BSEP: bile salt export; Caco-2: permeability in an *in vitro* cellular permeability assay with Caco-2 cells; clogP: logarithm of compound partition coefficient between n-octanol and water; CPA: cyclopiazonic acid; HBA: number of hydrogen bond acceptors; HBD: number of hydrogen bond donors; HIA: human gastrointestinal absorption; HOB: human oral bioavailability; I: Toxicity category I is Highly toxic and Severely irritating; MW molecular weight; n-ROTB: number of rotatable bounds; OATP1B1 (organic anion transporting polypeptide 1B1 pharmacokinetic transporter; OATP1B3: organic anion transporting polypeptide 1B3 pharmacokinetic transporter; OATP2B1: organic anion transporting polypeptide 2B1 pharmacokinetic transporter; PPB: plasma protein binding; P-gp: P-glycoprotein; Po/w: n-octanol/water partition coefficient; TPSA: polar surface area. Obtained from admetSAR version 2; http://lmmd.ecust.edu.cn/admetsar2/ (accessed on 12 September 2024).

## Data Availability

The original contributions presented in the study are included in the article, further inquiries can be directed to the corresponding author.

## Supplementary Materials

The following supporting information can be downloaded at: https://www.mdpi.com/article/10.3390/toxins16110473/s1, File S1: Figure S1 (a): Cytotoxic effects of CPA in 3D SH-SY5Y spheroids by MTT assay. Data are expressed as mean ± SEM of three independent experiments (*n* = 3). (*) *p* ≤ 0.05 indicates a significant difference with respect to the control; Figure S1 (b): Cytotoxic effects of CPA in 3D SH-SY5Y spheroids by ATP assay. Data are expressed as mean ± SEM of three independent experiments (*n* = 3). (*) *p* ≤ 0.05 indicates a significant difference with respect to the control.
